# Next step in the development of mesoprogestins: the preclinical profile of EC313

**DOI:** 10.3389/fendo.2023.1201547

**Published:** 2023-09-08

**Authors:** K. Błaszczak-Świątkiewicz, A. Krupa, E. Mnich, W. Elger, M. Oettel, H. Nair, M. Wierzbicki, P. Sieroszewski, Z. Shaked

**Affiliations:** ^1^ R & D Centre, Evestra Onkologia Sp z o.o, Lodz, Poland; ^2^ Evestra, Inc., Corporate Headquarters, Schertz, TX, United States; ^3^ Department of Gynaecology and Obstetrics, Medical University of Lodz, Lodz, Poland

**Keywords:** mesoprogestins, selective progesterone receptor modulators, uterine fibroids/leiomyomas, endometriosis, progesterone receptor agonists and antagonists

## Abstract

**Introduction:**

The pharmacological target for progesterone, different progestins, and Selective Progesterone Receptor Modulators (SPRMs) is the nuclear progesterone receptor (PR). EC313 is a new member of a subgroup of SPRMs, mesoprogestins, which combine especially PR- agonistic and PR-antagonistic activities in one molecule.

**Methods:**

The suitable *in vivo*-model for the differentiation of SPRMs from the subgroup of mesoprogestins is the estrogen-primed juvenile rabbit endometrium assay (McPhail Assay). Remarkably, in contrast to other well-known SPRMs with no agonistic effects in this test, EC313 shows clear partial PR-agonistic effects that are higher than that of the well-known mesoprogestin Asoprisnil which already demonstrated remarkable clinical effectiveness for the treatment of uterine fibroids and endometriosis. The findings from the guinea pig studies presented here can be the impetus for further preclinical development of EC313. This model shows the same features for the termination of pregnancy by antiprogestins such as Mifepristone and Ulipristal acetate (UPA) in humans. Moreover, it is possible to distinguish between progestational and anti-progestational activities in the same experiment.

**Results:**

The EC313 treatment reveals PR dominance in the genital tract and inhibits unopposed estrogenic effects. In very high doses (30.0 mg/animal/day subcutaneously (s.c.)) given twice on pregnancy days 43 and 44, no premature labor was induced (in contrast to UPA, dosed at 10.0 and 30. mg/animal/day s.c.). The anti-ovulatory activity of EC313 exceeds that of Ulipristal acetate or Mifepristone. EC313 binds to the steroid receptors *in vitro* with a similar affinity as the natural ligand progesterone. At the glucocorticoid receptor (GR) EC313 acts as a weak inhibitor. Minor activities at the human androgen receptor (AR) and mineralocorticoid receptor (MR) are considered negligible. No binding to the estradiol receptor was detected. In contrast to some *in vitro*-receptor findings, estrogenic, anti-estrogenic, androgenic, anti-androgenic, glucocorticoid, and anti-glucocorticoid actions were absent *in vivo*. The tissue selectivity of EC313 was demonstrated previously by reducing the growth and proliferation of uterine fibroids in animal models (lowest effective dosage 0.1 mg/kg/day s.c.).. As shown in this article, the anti-fibroid activity of EC313 was confirmed with a 10 times lower dosage (0.01 mg/kg/day s.c.). It was also shown that EC313 reduces the growth of endometriotic lesions in a human xenograft immune-deficient (NOD-SCID) mice model with a comparatively very low dosage range. In the aforementioned EC313 activity model, UPA was tested as the reference compound, the clinical effectiveness of which has already been demonstrated.

**Discussion:**

For an explanation of these findings, the possibility is discussed that the mixed agonistic/antagonistic feature of EC313 is tissue target-specific based on its super-additive synergism characteristic for active bifunctional agents. In conclusion, the specific pharmacodynamic profile of this compound opens the possibility for the development of a drug with a distinct pharmaco-endocrinological profile against uterine fibroids, endometriosis, and other PR-dependent gynecological diseases.

## 1 Introduction

The modulation of suitable steroid-receptor activities opened complete new therapeutic approaches as shown by Selective Estrogen Receptor Modulators (SERMs) (1–5). The functions of progesterone receptors (PRs) play a vital role in the structure, function, and regulation of the female reproductive tract, including pregnancy (6). In men, progesterone is essential, e.g., for spermiogenesis, CNS functions, and prostate pathology (7). Selective Progesterone Receptor Modulators (SPRMs) influence important progesterone-regulated pathways. They may exert an antagonistic, agonistic, or mixed response (7).

The history of SPRMs started in the 1980s when mifepristone (RU486) was discovered. Since then several SPRMs have been developed. Two of them are in current clinical use: Mifepristone for pregnancy interruption and Ulipristal acetate (CDB-2914) for managing abnormal uterine bleeding (AUB) in women with uterine leiomyomas (fibroids) and as an emergency contraceptive in higher dose (8).

Besides female health, other new clinical indications have been discussed such as osteoporosis via the receptor activator of nuclear factor kappa B (NFκB) and its ligand RANKL (9, 10) and hyperpigmentation disorders via a unique mechanism that encompasses a selective inhibition of melanosome export (11).

Despite great efforts, a perfect SPRM does not yet exist (12). Most of the known SPRMs show moderate mixed antagonistic/agonistic actions in which the antagonistic profile is dominant. However, the agonistic action was not seen in the rabbit endometrium for those compounds. The exception is asoprisnil which was proved to have more PR-agonistic than -antagonistic features in the rabbit endometrium (13). For the subgroup of SPRMs steroids with an Asoprisnil-like profile, the term “mesoprogestins” was created (14). Unfortunately, the typical human endometrial changes under SPRM therapy—progesterone receptor modulator associated endometrial changes (PAEC)—were misinterpreted during the clinical development of Asoprisnil and, as a result, further development of asoprisnil was stopped. It should be noted that PAECs were finally recognized as benign, not related to cancer, not precancerous, and reversible (15, 16).

It is an unmet medical need to optimize the mesoprogestin strategy to find compounds with higher PR-agonistic partial effects without the loss of PR-antagonistic properties. One of these SPRMs is EC313 of which the following preclinical endocrine-pharmacological profile is presented.

## 2 Materials and methods

### 2.1 Synthesis of test substance EC313

**Figure 1:**
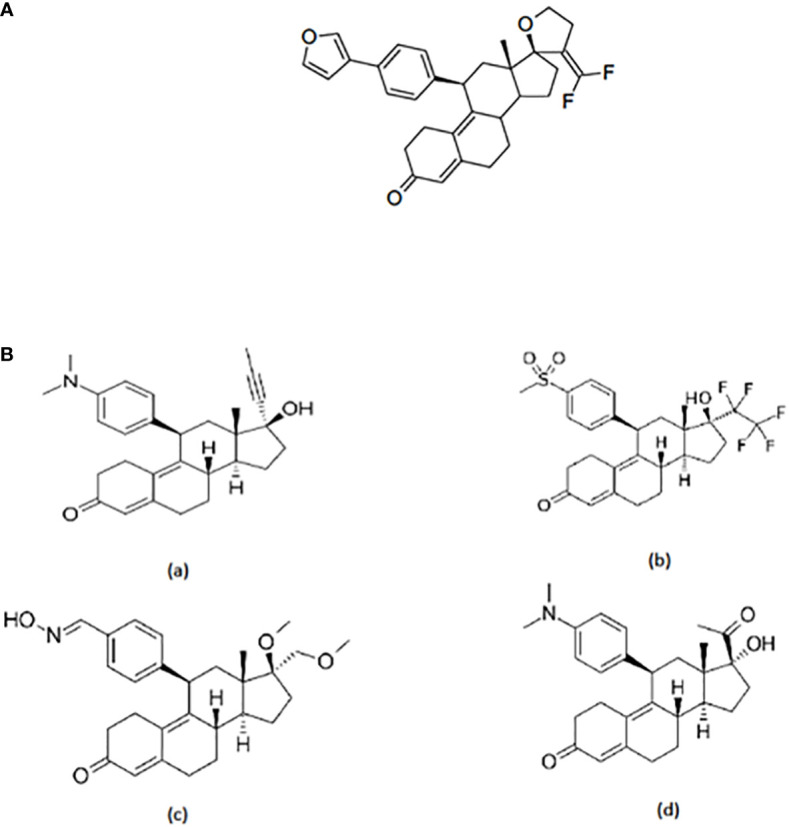
**(A)** Structure of EC313. 21,21-difluoro-11β-[4’-(3’-furanyl)phenyl]-17,23-epoxy-19,24-dinor-17α-chola-4,9,20-triene; molecular formula C32H32O3F2; mol. weight 502,59. **(B)** Structure of selected SPRMs: (a) Miferistone, (b) Vilaprisan, (c) Asoprisnil, and (d) Ulipristal acetate.

A series of antiprogestins have been synthesized by partially fluorinating the steroid molecule in positions relevant to receptor binding. By introducing fluorine at the exo-methylene of the 17 spirofuran ring EC313 has been synthesized (17) (**Figure 1A**).

The structure of EC313 clearly differs from other SPRMs such as Mifepristone, Vilaprisan, Asoprisnil, and UPA (**Figure 1B**).

EC313 is a steroid with very low aqueous solubility. Since the calculated log Po/w is > 7, therefore, the presented studies were performed with an oily solution as the vehicle (see details in the *in vitro* and *in vivo* studies sections).

### 2.2 *In vitro* studies

#### 2.2.1 Chemicals and reagents

##### 2.2.1.1 Cell culture

All the cell lines, CHO (Chinese hamster ovaries), U2OS (human osteosarcoma), and HELA (human cervix epitheloid carcinoma) were purchased from Sigma-Aldrich.

##### 2.2.1.2 Plasmids

individual cDNA encoding full length of human steroid receptor was subcloned to pcDNA3.1+/C-(K)-DYK(mammalian) vector and delivered by Genscript. Vector GL4.26[*luc2*/minP/Hygro] coding the estrogen responsive element (ERE) was prepared by Genscript. Vector pGL4.36[*luc2P*/MMTV/Hygro] was purchased in Promega.

##### 2.2.1.3 Cell culture medium composition

for the CHO and U2OS cell lines – RPMI 1640 phenol red free with 10% FBS (charcoal stripped), L-Glutamine; for HELA cell line - medium EMEM phenol red free with 10% FBS (charcoal stripped), sodium pyruvate, L-Glutamine, NEAA (purchased in Sigma, BioWest and Thermo Fisher Scientific, respectively).

##### 2.2.1.4 Chemicals

the test article, EC313, and Vilaprisan were obtained from Evestra INC, and other test reference compounds, Promegestone, Mifepristone, Dexamethasone, Flutamid, Aldosterone, Spironolactone, Estradiol, and Fulvestrant, were purchased from Sigma Aldrich and metribolone in Advanced Chem Block. *Transfection reagents*: FuGENE and Opti-MEM medium were delivered by Promega and Thermo Fisher Scientific, respectively.

#### 2.2.1 Competitive binding to human nuclear receptor proteins

The study aimed to characterize the competitive binding profile of EC313 to relevant human nuclear receptors. For the assays, commercially available ‘ligand binding domain’ proteins were purchased (Thermo Fisher Scientific). In addition, the full-length receptor proteins were expressed in transfected HI insect cells using the `Bac-to-bac Baculovirus expression system´ (Thermo Fisher Scientific). Cellular lysates served as receptor preparation. Receptor protein preparations were incubated with tritiated reference ligands and EC313 or reference steroids at concentrations between 0.1 and 1000 nM for 2h at 4°C to reach equilibrium. Subsequently, unbound steroids were adsorbed to dextran-coated charcoal (Sigma Aldrich), separated by filtration, and the remaining radioactivity was quantified in a ß-counter (Perkin Elmer). Displacement curves were fitted using the GraphPad Prism 7 free version to obtain EC50 values. Relative binding affinity (RBA [%], (C50ref/C50test)*100) and the competition coefficient (C50test/C50ref) were used to describe the relative binding potency of EC313 compared to the reference ligands.

#### 2.2.2 Steroid hormone receptor transactivation

The ability of EC313 to transactivate steroid hormone receptors characterizing the agonistic or antagonistic profile of the compound at the level of different steroid hormone receptors *in vitro* is listed in **Table 1** together with the methodological details of the transactivation experiments.

**Table 1:** Cellular test systems and transfected plasmids employed in the reported assays.

Receptor	Cell Line	1^st^ Transfection Human Receptor cDNA	2^nd^ Transfection: Reporter Gene Construct	Reference Agonist	Reference Antagonist
**PRA**	CHO	pcDNA 3.1hPRAstable transfection	pGL4.36-MMTV-LUC transient transfection	Promegestone	Mifepristone
**PRB**	CHO	pcDNA 3.1hPRBstable transfection	pGL4.36-MMTV-LUC transient transfection	Promegestone	Mifepristone
**Erα**	CHO	pcDNA 3.1hERtransient transfection	pGL4.26-ERE-LUC transient transfection	Estradiol	Fulvestrant
**ERβUnspecific Response**	CHO	pcDNA 3.1hERβ	pGL4.26-ERE-LUC	Estradiol	Fulvestrant
**AR**	CHO	pcDNA 3.1ARtransient transfection	pGL4.36-MMTV-LUC transient transfection	Metribolone	Flutamide
**MR**	U2OS	pcDNA 3.1MRtransient transfection	pGL4.36-MMTV-LUC transient transfection	Aldosterone	Spironolactone
**GR**	HELA	NONEGR is constitutively expressed	pGL4.36-MMTV-LUC stable transfection	Dexamethasone	Mifepristone

#### 2.2.3 Transactivation characteristic of EC313

The expression of tested human steroid receptors upon EC313 influence was analyzed on the transcriptional level. The analyses of the sensitivity of the transactivation test systems were performed in a variety of cellular systems so as to study the significant response of the desirable steroid receptor. The specificity of the test system relied on the expression of the LUC reporter gene construct under the control of a promoter responsive to the transfected receptor. Appropriate cell lines carried either stable or transient expressions of the respective steroid receptor.

##### 2.2.3.1 Progesterone receptor gene expression analysis

Due to the existence of two isoforms of the PR receptor (PRA and PRB) and the fact that both of them control expression of a largely different set of genes, separate assays for each of them were carried out. The ability of EC313 to induce/inhibit transactivation of PRA and PRB was studied together with Promegestone (R-5020) serving as the reference agonist and Mifepristone as the antagonist.

##### 2.2.3.2 Plasmid generation

In order to develop the transfection assay, plasmids were generated and isolated. Plasmids included vectors with the coding sequences for the receptor as well as the reporter gene, respectively. The vector pcDNA3.1 coding sequence for the full length of human PRA and PRB (cDNA ORF clone in pcDNA3.1+/C-(K)-DYK) was used as an expression vector for PR isoform A and B, respectively. Vector pGL4.36 coding for the progesterone responsive element MMTV and firefly luciferase reporter vector [luc2P/Hygro] were used as reporter systems (pGL4.36-MMTV/LUC).

##### 2.2.3.4 Cell line test system for PR

A sufficient transactivation signal was achieved using stable transfection for the human PRA and PRB receptors and transient transfection for the MMTV-luciferase reporter gene. Therefore, the first stable transfection with the receptor of interest was performed. Thus, 0.5x10^6^ CHO cells per well were plated into 6-well plates in transfection medium w/o antibiotics (RPMI phenol-free, 10% FBS,2mM L-glutamine). When the cells reached 80% confluency, transfection with receptor using FuGENE 6 in Opti-MEM medium was accomplished according to the manufacturer’s instruction. After 48h of transfection, stably transfected cells were cultured in a growth medium containing an antibiotic (G418 500 µg/mL). Only cells with the resistance gene (resistance to G418), which had been transfected together with the DNA for PRA and PRB were able to grow in this medium. Consequently, growing cell clones were selected for further steps as stable transfectants. The CHO cells stably transfected with the expression of PRA or PRB were transiently transfected with promoter MMTV/LUC vectors. For this purpose, cells with a density of 12.5x10^3^ per well in a 96-well plate were transfected using transfection reagent FuGENE and Opti-MEM medium, following the manufacturer’s instructions. The recovery of cells lasted 24h and they were then moved into the culture medium and treated for 24h with test compounds. 

The capacity of EC313 to induce/inhibit the tested receptors was studied in agonistic and antagonistic approaches. To determine the agonistic activity, EC313 was tested in the concentration range 0.009 nM - 1200 nM and its activity was compared with Promegestone tested in the corresponding concentration range (0.009 nM - 1200 nM). The antagonistic activity was assessed during the competitive interaction between EC313 (0.009 nM - 1200nM) and the appropriate concentration of Promegestone for PRA or PRB, respectively (**Table 2**). As a reference, Mifepristone (0.009 nM - 1200 nM) was used. After 24h incubation of cells with tested compounds, luciferase activity was measured using a VICTOR™ X Multilabel Plate Reader -Perkin Elmer.

**Table 2:** Reference compound concentration for antagonistic activity.

CONCENTRATION FOR ANTAGONISTIC ACTIVITY		
	Promegestone for PRA [nM]	Promegestone for PRB [nM]
Stably transfected cells	35	1.0

##### 2.2.3.5 Glucocorticoid receptor gene expression analysis

HELA cells line carrying endogenous glucocorticoid protein were cultured in order to study gene expression on the transcriptional level under the influence of the tested compounds. The cells were transfected with reporter vector MMTV/LUC. To produce high protein overexpression, stable transfection was carried out. Next, the transfected HELA cells expressing glucocorticoid receptor and reporter vector MMTV/LUC were stimulated by the tested compounds (Dexamethasone or EC313) or the mixture: Dexamethasone and an appropriate range of concentration of EC313. Agonist and antagonist properties of EC313 toward tested receptor GR were analyzed using luminescence detection.

##### 2.2.3.6 Plasmid generation

In order to develop the transfection assay, plasmids were generated and isolated. Vector pGL4.36 coding the glucocorticoid receptor responsive element MMTV and firefly luciferase reporter vector [luc2P/Hygro] were used as a reporter system (pGL4.36-MMTV/LUC). The cells were transfected with reporter vector MMTV/LUC.

##### 2.2.3.7 Cell line test system for GR

stable transfection with reporter vector was performed on a 6-well plate (0.5x10^6^ cells per well) in culture medium (EMEM, 10% FBS, NEAA, sodium private, L-glutamine). Briefly, according to the transfection procedure, cells were passaged, and they grew in a culture medium containing an appropriate concentration of the antibiotic (Hygromycin 150 µg/mL). Resistance to Hygromycin gene, which had been transfected together with the reporter vector to the cells, only allowed positively transfected cells to grow in this medium. Therefore, growing cell clones were selected for further steps as stable transfectants.

The agonistic profile of EC313 was tested in the concentration range 0.009 nM - 1200 nM together with Dexamethasone tested in the corresponding concentration range (0.009 nM - 1200 nM). The antagonistic profile was assessed during the competitive interaction between EC313 (0.009 nM – 1200 nM) and 11.5 nM of Dexamethasone for stable transfection. As a reference system, mifepristone (0.009 nM - 1200 nM) was used. After 24h incubation of cells with the tested compounds, luciferase activity was measured using VICTOR™ X Multilabel Plate Reader -Perkin Elmer.

##### 2.2.3.8 Androgen receptor gene expression analysis

The androgen transactivation potential of EC313 was studied in the CHO cell line expressing this steroid protein receptor. The cells were co-transfected with a vector encoding a human full-length androgen receptor (hAR) linked to the reporter vector MMTV/LUC. To produce high protein expression, transient transfections were carried out. Next, co-transfected CHO cells were stimulated by the tested compounds (Metribolone or EC313) or the mixture: Metribolone and an appropriate range of concentrations of EC313. Agonist and antagonist properties of EC313 toward tested receptor AR were analyzed using luminescence detection.

##### 2.2.3.9 Plasmids generation

To prepare the transfection assay, plasmids were generated and isolated. Plasmid included vector coding sequences for the receptor as well as the reporter gene. Vector pcDNA3.1 coding sequence for the full length of human AR (AR cDNA ORF clone in pcDNA3.1+/C-(K)-DYK) was used as an expression vector for AR. Vector pGL4.36 coding for the androgen-responsive element (MMTV) linked to Firefly luciferase [luc2P/Hygro] was used as a reporter system (pGL4.36-MMTV/LUC).

##### 2.2.3.10 Cell line test system for AR

Sufficient transactivation signal was achieved using transient transfection for the human AR receptor and transient transfection for the MMTV-luciferase reporter gene thus transactivation assay was performed only for transiently co-transfected cells. Co-transfection was performed on a 96-well plate (16x10^3^ cells per well) in culture medium (RPMI w/o phenol red, 10% FBS, L-Glu) w/o antibiotics using transfection reagents following the manufacturer’s instructions. Prior to transfection, plated cells were approximately 70%–80% confluent. After 24h of transfection, medium was removed to a culture medium and cells were treated for the next 24h with the test compounds.

The agonistic profile of EC313 (0.009 nM - 1200 nM) was analyzed in comparison to Metribolone (Methyltrienolone, R 1881) (0.009 nM - 1200 nM). The antagonistic activity was detected based on the competitive interaction between EC313 (0.009 nM - 1200nM) and 13nM Metribolone. To reflect the potency of EC313, Flutamid (0.009 nM - 1200 nM) and 13 nM Metribolone were tested as a reference system. After incubating the cells for 24h with the tested compounds, the luciferase activity was analyzed.

##### 2.2.3.11 Mineralocorticoid receptor gene analysis

The U2OS cell line which expresses the mineralocorticoid receptor was used to study the gene expression on the transcriptional level under the influence of EC313 and the reference compound. The cells were transfected with a vector encoding a human full-length mineralocorticoid receptor (hMR) linked to the reporter vector MMTV/LUC. Cells were transiently and stably transfected in order to reach high protein expression. Next, co-transfected U2OS cells carrying mineralocorticoid receptors and reporter vector MMTV/LUC were stimulated by the tested compounds (Aldosterone or EC313) or the mixture: aldosterone and an appropriate range of concentrations of EC313. The agonist and antagonist properties of EC313 toward tested receptor MR were analyzed using luminescence detection.

##### 2.2.3.12 Plasmid generation

In order to develop the transfection assay, plasmid-included vectors coding sequences for the receptor as well as the reporter gene were generated and isolated, respectively. Vector pcDNA3.1 coding sequence for full length of human MR (MR cDNA ORF clone in pcDNA3.1+/C-(K)-DYK) was used as an expression vector for MR and was linked to the reporter system (pGL4.36-MMTV/LUC) building with vector pGL4.36 coding the mineralocorticoid responsive element (MMTV) and firefly luciferase [luc2P/Hygro].

##### 2.2.3.13 Cell line test system for MR

Since sufficient transactivation signal was achieved using transient transfection for the human MR receptor and transient transfection for the MMTV-luciferase reporter gene, therefore, a transactivation assay was performed for transiently co-transfected cells. Transfection was performed on a 96-well plate (15.5x10^3^ cells per well) in culture medium (RPMI phenol red, 10% FBS, L-Glu) w/o antibiotics according to the manufacturer’s specification. Prior to transfection, plated cells were approximately 70%–80% confluent. 24h later, cells were treated with test compounds overnight.

The properties of EC313 to induce/inhibit tested receptor was studied in agonistic and antagonistic approaches. Co-transfected U2OS cells were stimulated with either aldosterone (0.009 nM - 1200 nM) or EC313 (0.009 nM - 1200 nM) in order to determine and compare the agonist potential of test compounds. The antagonistic activity was checked in competitive interaction between EC313 (0.009 nM - 1200 nM) and 0.4 nM of Aldosterone. The reference system, Spironolactone (0.009 nM - 1200 nM) and 0.4 nM Aldosterone, was tested as well.

##### 2.2.3.14 Estrogen receptor gene analysis

The CHO cell line was selected to express estrogen α and β protein and the gene expression under the tested compounds stimulation was analyzed. The co-transfection with vector encoding the human full-length steroid receptors hERα and hERβ and reporter vector ERE/LUC was performed. Transient and stable transfectants with protein overexpression were induced by tested compounds (17β-estradiol or EC313) or the mixture: estradiol and appropriate range of concentration of EC313. Agonist and antagonist properties of EC313 toward tested receptor ERα and ERβ were analyzed using luminescence detection.

##### 2.2.3.15 Plasmid generation

Plasmid included vectors coding sequences for the receptor as well as the reporter gene. The vector pcDNA3.1 coding sequence for the full length of human ERα or ERβ (cDNA ORF clone in pcDNA3.1+/C-(K)-DYK) was used as an expression vector for the appropriate protein. Vector pGL4.26 coding for the estrogen responsive element (ERE) and firefly luciferase [luc2/minP/Hygro] was used as a reporter system (pGL4.26-ERE/LUC).

##### 2.2.3.16 Cell line test system for ER

Since sufficient transactivation signal was achieved using transient transfections for both the human ERα and ERβ receptors and ERE-luciferase reporter gene, the transiently co-transfected cells were used in the test. Transfection was performed on a 96-well plate (12.5x10^3^ cells per well) in culture medium (RPMI w/o phenol red, 10% FBS, L-Glu) w/o antibiotics. The ratio of DNA to transfection reagent was 1:3 as described in the manufacturer’s information. Only confluent cells were stimulated by the test compounds over 24h.

The ability of EC313 to agonize and/or antagonize ER receptor expression was analyzed in the following test system: co-transfected CHO cells were stimulated with estradiol (0.009 nM - 1200 nM) and EC313 (0.009 nM - 1200 nM) for detection agonistic activity. The antagonistic activity was assessed during the competitive interaction between EC313 (0.009 nM - 1200nM) and an appropriate concentration of estradiol (see **Table 3**). Fulvestrant (0.009 nM - 1200 nM) was used as the reference system. After incubating the cells for 24h with the tested compounds, the luminescent signal generated by the luciferase activity was detected. 

**Table 3:** Reference compound concentration for antagonistic activity.

CONCENTRATION FOR ANTAGONISTIC ACTIVITY		
	Estradiol for ERα [nM]	Estradiol for Erβ [nM]
Transiently transfected cells	25	1.5

Graphs with representative results were plotted using the free version of the GraphPad Prism 7 software. Half-maximal effective concentration (EC50) and half-maximal inhibitory concentration (IC50) were determined in dose-response correlation. Each set of experiments was performed in triplicate and data were presented as mean value ± standard deviation (SD).

#### 2.4.1 Additional studies

##### 2.4.1.1 Induction of progesterone receptor nuclear translocation by EC313

T47D cells were treated with EC313 and a range of reference ligands (i.e., Ulipristal acetate, Asoprisnil, Mifepristone, Estradiol, and Promegestone) at a concentration of 100 nM for 6h and 24 hours to activate the PR. Subsequently, progesterone receptors present in nuclear as well as cytosolic fractions were stained using Western blotting.

##### 2.4.1.2 Investigation of PR-B – c-SRC interaction induced by EC313 using a two-hybrid system

After the luciferase assay, the CHO cells were plated on a 96-well plate and transfected with pG5, pBIND-c-SRC, and pACT-hPR-b. 24h after the transfection, tested and reference compounds were added in a concentration range of 10^-8^ to 10^2^ nM in a hormone-free medium and incubated for 24h. After the treatment, the medium was decanted from cells and Lysis buffer was added followed by an addition of Dual-Glo- Reagent I. Plates were incubated for 10 min. The firefly luciferase activity was measured on plate reader VICTOR™ X, Perkin Elmer. To measure the Renilla luciferase activity, Dual Glo Reagent II was added and plates were read once again. The data were analyzed to obtain EC50 and values using GraphPad Prism 7. Each compound was tested in triplicate in a single experiment and all assays were performed five times.

##### 2.4.1.3 Investigation of PRB – NCoR interaction induced by EC313 using a two-hybrid system

The unstimulated progesterone receptor is bound to chaperones such as heat shock proteins and resides largely in the cytoplasm. Upon activation by a ligand, the chaperones are shed and the receptor can dimerize, interact with other proteins, and translocate to the nucleus.

T470 cells (constitutively expressing the progesterone receptor) were treated with 100 nM of EC313 or reference compounds to activate PR. After 6h and after 24h cells were lysed, the nuclear and cytosolic fractions were separated and Western blots of these fractions were prepared.

Blots were stained for PRA and PRB as well as the housekeeping proteins GAPDH (as a marker for the cytosolic fraction) and histone H3 (as a marker for the nuclear fraction). Nuclear and cytoplasmic proteins from 1470 cells were extracted after cell stimulation with PR ligands and the nuclear and cytoplasmic fractions were analyzed for PRB and PRA content using Western blotting.

### 3.2 *In vivo* studies

All experiments were performed under the Institutional Guideline which recommends that all procedures for animal care and use be documented and in compliance with regulatory standards. The recommended environmental conditions include a temperature of 22 ± 3°C; relative humidity of 30%-70% (preferably 45%-65%), adequate ventilation (15-20 cycles of air exchange per hour), and a 12-hour light/dark cycle. Group housing of two or three rats per cage is preferred, in a cage of approximately 2000 cm^2^, and 1-3 rabbits in a cage of approximately 5456 cm^2^. Laboratory diet and water are typically provided ad libitum. The rats (Wistar CRL WI), rabbits (White New Zealand), and guinea pigs (Dunkin-Hartley) used in our experiments were delivered from Charles River (Germany) and had the required institutional animal health certifications. The relevant consent of Local Ethics Committees for animal testing has been obtained.

#### 32.1 .Guinea pig studies

The guinea pig may be used as a model for the effects of substances on the motor functions of the human uterus (data in press). As in humans, RU486 (mifepristone) also induces abortion or labor in advanced pregnancy in guinea pigs (18).

##### 3.2.1.1 Determination of abortive activities

In female animals, the second day of vaginal opening in the mating cycle was defined as day 1 of pregnancy (pregnancy length approx. 62 days). They were treated on days 43 and 44 by subcutaneous (s.c.) injection of the proprietary compounds EC312, EC313, EC323, and EC328 as well as UPA. All tested compounds were synthesized in Evestra Inc. The dose levels administered were 3, 10, and 30 mg/animal/day. The external genital organs were inspected for the presence of vaginal bleeding and fetal membranes (twice daily). The presence of expelled fetuses and placentae in the cages was also recorded at each inspection.

The animals were sacrificed on day 50 using CO_2_. Both uterine horns were dissected and their contents were inspected. Fresh corpora lutea (CL) were evidence of a recent ovulation.

##### 3.2.1.2 Determination of ovulation inhibition

Test compounds were administered once daily from cycle days 10-17 by s. c. injection using 0.2 mL vehicle (benzyl-benzoate/castor oil, ratio 1 + 4 v/v). The time of autopsy (day 18) was 24-48h after the expected ovulation at the end of the treatment cycle. This design permits the study of the effects of the tested compounds on the expected ovulation. Fresh CL were evidence of a recent ovulation.

#### 3.2.2 PR-Activity on endometrial transformation in rabbits (McPhail assay)

The secretory transformation of estrogen-primed endometrium of immature rabbits indicates the strength of the progestagenic action on the endometrium of the chosen test compound. The simultaneous administration of test compounds with progesterone can indicate potential anti-progestagenic actions.

Female rabbits received daily s.c. injections of 5.0 µg 17β-Estradiol/per animal for 6 days leading to the proliferation of the endometrium. From day 7 to day 13 the animals received doses of vehicle, EC313 (1 to 30 mg/animal/day s.c) or progesterone. On day 14, the animals were sacrificed and the uteri were processed for histological evaluation and the secretory endometrial transformation was rated according to a modified McPhail score (1 – 4).

#### 3.2.3 Glucocorticoid and anti-glucocorticoid activity in rats

The standard Thymus-Involution-Test in rats was used. Adrenalectomized male Wistar Rats were treated for 4 consecutive days with EC313 s.c. (10, 30, and 100 mg/animal/day), with dexamethasone alone or simultaneously with EC313 and dexamethasone s.c. (0.1 mg/animal/day). On day 5 the animals were sacrificed, and the thymus and spleen weights were determined. Reduced weight of the thymus indicated glucocorticoid activity. Stopping the dexamethasone-induced thymus reduction by the test compound indicated anti-glucocorticoid activity.

#### 3.2.4 Androgenic and anti-androgenic activity in rats (Hershberger assay)

To test EC313 for potential androgenic or anti-androgenic potential, the Hershberger assay was used. Orchiectomized male Wistar rats were treated for 7 consecutive days with daily subcutaneous injections of EC313 (3, 10, and 30 mg/animal/day).

The potential anti-androgenic activity was assessed by co-administration of Testosterone propionate (TP; 1 mg/animal/day s.c.), which also served as the positive control, and EC313. The vehicle served as the negative control. One day after the last treatment, animals were sacrificed and the prostate gland and the seminal vesicles were removed and weighed. The relative weight of these organs, expressed as mg/100 g body weight, served as the endpoint.

#### 3.2.5 Estrogenic and anti-estrogenic activity in rats

For determination of estrogenic effects immature female ovariectomized Wistar rats were treated for 3 days with high doses of EC313 (20 and 50 mg/animal/day) or the positive control 17β- Estradiol (E2) at 1.5 µg/animal/day. The vehicle served as the negative control. After treatment, the animals were sacrificed on day 4, and their vagina and uterine horns were dissected and weighed.

#### 3.2.6 Humanized endometriotic xenografts in mice

Endometriosis is one of the expected clinical target indications for the development of EC313. But, there are no reliable pre-clinical models known at present. From all experimental approaches, the transplantation of human endometriotic tissue into immunodeficient mice resembles the clinical situation to some extent closely. Treatment of EC313 was compared with UPA as a reference compound. Two to four small pieces of human endometriotic tissue were placed into a s.c. pouch in the pelvic area of each of the ovariectomized mice. At the same time, an E2-releasing pellet (0.36 mg/pellet/60 days) was implanted s.c. in the neck of the mice. Furthermore, 2 weeks after implantation, the mice were treated with once-daily s.c. injections of vehicle, EC313 (0,01, 0.1, and 1 mg/animal/day), or Ulipristal acetate (1 mg/animal/day) for 14 days. The mice were sacrificed 24 hours after the last injection and all identified endometriotic grafts were resected, weighed, and subjected to visual examination.

#### 3.2.7 Humanized uterine fibroid xenografts in mice

Fibroids/Leiomyoma are one of the clinical target indications for the development of EC313. But, there are no very reliable pre-clinical models known at present. From all experimental approaches, the transplantation of human fibroid tissue into immunodeficient mice s.c. resembles the clinical situation most closely. UPA served as a reference compound. There were two study parts: A and B. Part A is already published (2). Human uterine fibroid samples (2x2x2 mm; weighting roughly 8 mg) were transplanted s.c. into NOD-SCID mice 3 weeks after ovariectomy. Simultaneously, the animals were supplemented s.c. with E2-releasing pellets (0.05 mg E2/90 days release). The treatment period over 60 days (5 treatment days a week) started immediately after transplantation. There was a sham control, an E2 control, two EC313-group, one Ulipristal (UA) group, and a combination of UPA+EC313 (Part A). In Part B, the animals in the treatment groups were dosed with EC313 doses of 0.1 and 1.0 mg/kg/day for confirmation of previous findings. The animals were sacrificed after a treatment period of 60 days. Graft weights were the primary endpoint (parts A and B); histological findings including immunostainings of the grafts were used as secondary endpoints (part A).

## 4 Results

### 4.1 *In vitro* studies

#### 4.1.1 Competitive binding assay

##### 4.1.1.1 Competitive binding to the human progesterone receptor

**Figure 2:**
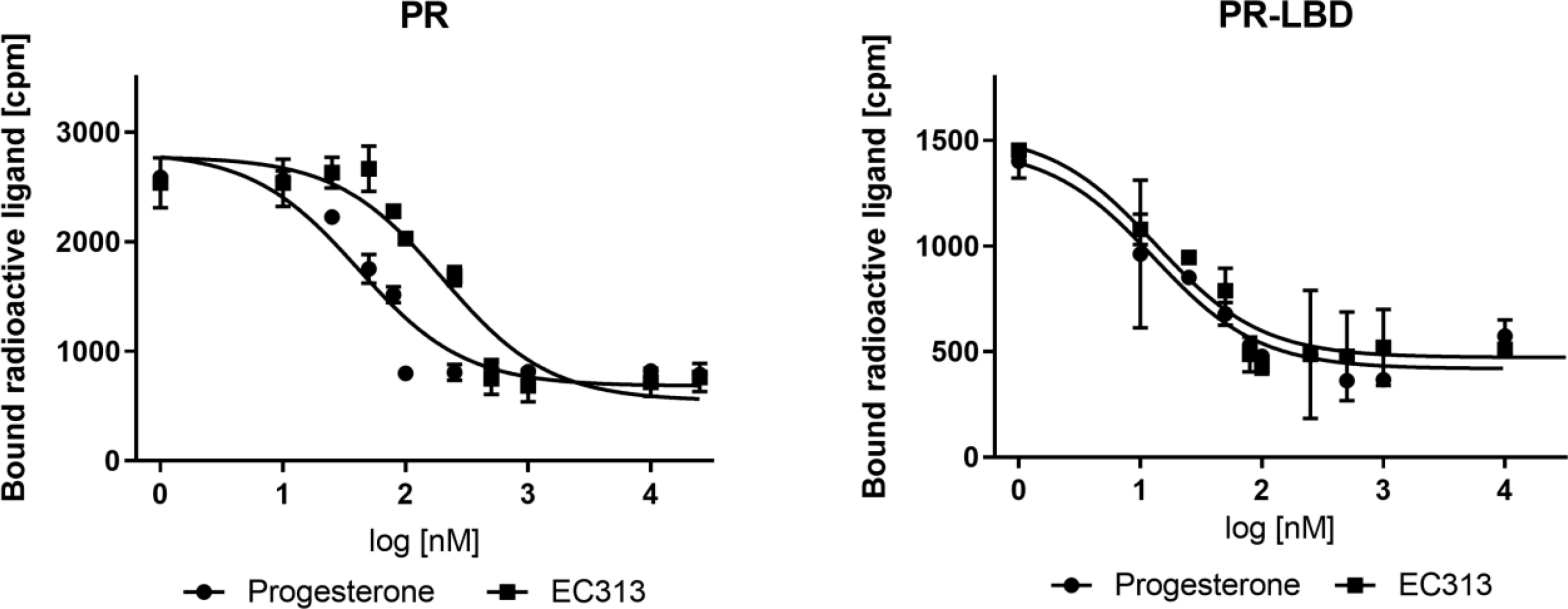
Competitive binding assay curve, binding of EC313 and progesterone to PR and PR-LBD.

The results of four independent experiments demonstrated that EC313 binds competitively to the progesterone receptor and its ligand binding domain. Its affinity to the ligand binding domain was the same as that of progesterone. At the full length receptor, EC313 was slightly less potent (competition coefficient 3.4) compared to the natural ligand (**Figure 2**).

##### 4.1.1.2 Competitive binding to the human glucocorticoid receptor

**Figure 3:**
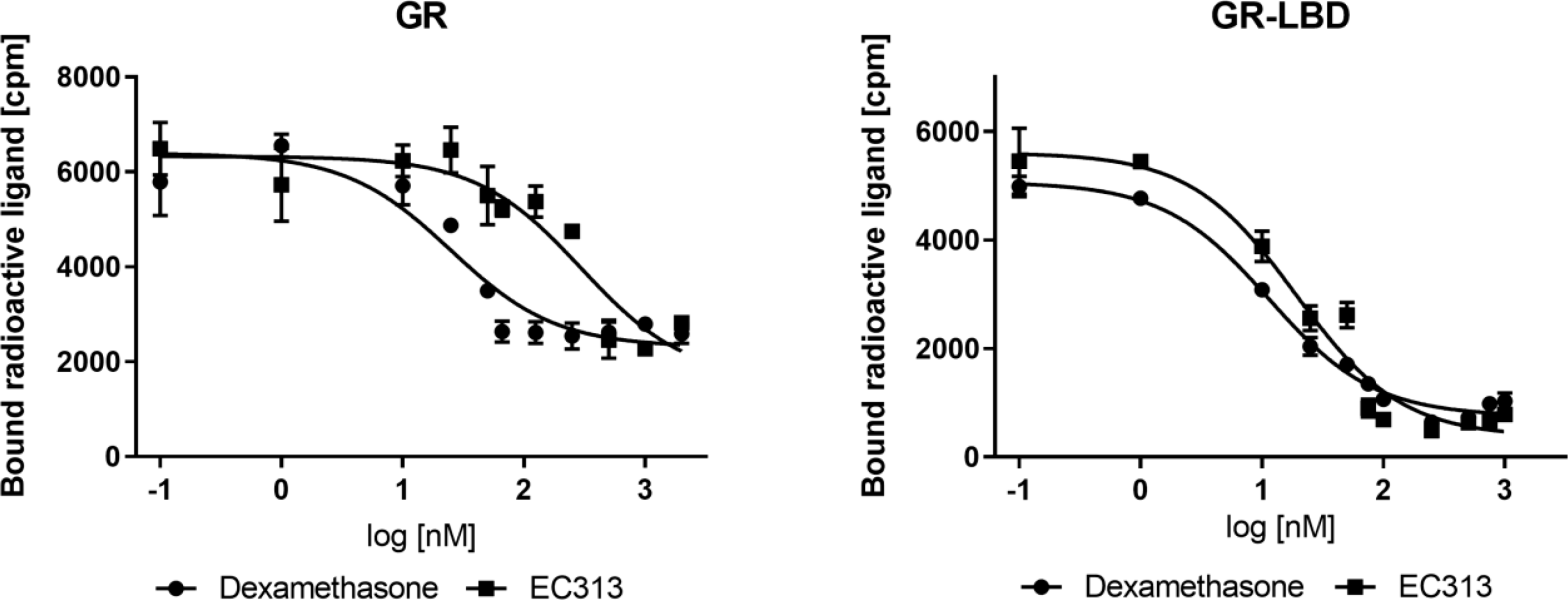
Competitive binding assay curve, binding of EC313 and dexamethasone to GR and GR-LBD.

EC313 competitively binds to the ligand binding site of the human glucocorticoid receptor with a similar affinity as dexamethasone. At the full length receptor, EC313 was more than an order of magnitude less affine than the reference (competition coefficient 12) (**Figure 3**, **Table 4**).

**Table 4:** IC50, RBA, and competition coefficient of EC313 and Dexamethasone for glucocorticoid receptor and ligand binding domain.

hGR	GR		hGR-LBD purified recombinant protein	GR-LBD	
	EC313	Dexamethasone		EC313	Dexamethasone
**IC50 [nM]**	226.0 ± 9.0	19.2 ± 7.6	**IC50^1^ [nM]**	19.1 ± 0.9	11.4 ± 1.0
**RBA [%]**	8.5		**RBA [%]**	60	
**Comp. Coefficient**	11.8		**Comp. Coefficient**	1.7	

##### 4.1.1.3 Competitive binding to the human androgen receptor

For testing the relative binding affinity of EC313 to the androgen receptor, the purified ligand binding domain of the human receptor was employed. Expression of the full length, functional, human androgen receptor had not been successful, despite many attempts to optimize the process. These introduced modifications included a wide range for the multiples of infection, protein expression time, and, finally, changing from the Hi5 to Sf9 cell line. None of these modifications, however, resulted in a sufficient expression of the functional receptor protein.

**Figure 4:**
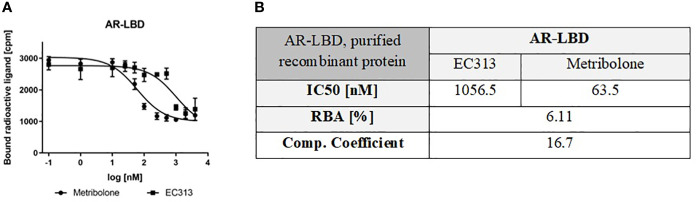
**(A)** Competitive binding assay curve, binding of EC313 and Metribolone to AR-LBD, **(B)** IC50, RBA and competition coefficient of EC313 and Metribolone for the ligand binding domain of the human androgen receptor.

In a series of experiments, the affinity of EC313 is found considerably lower than that of Metribolone with a competition coefficient of 17. Despite the fact that the generally less discriminative ligand binding domain of the androgen receptor was tested, EC313 demonstrated a low affinity to the protein. One may assume that the affinity to the full length receptor may be even lower (**Figure 4**).

##### 4.1.1.4 Competitive binding to the human estrogen receptors α and β-subtypes

**Figure 5:**
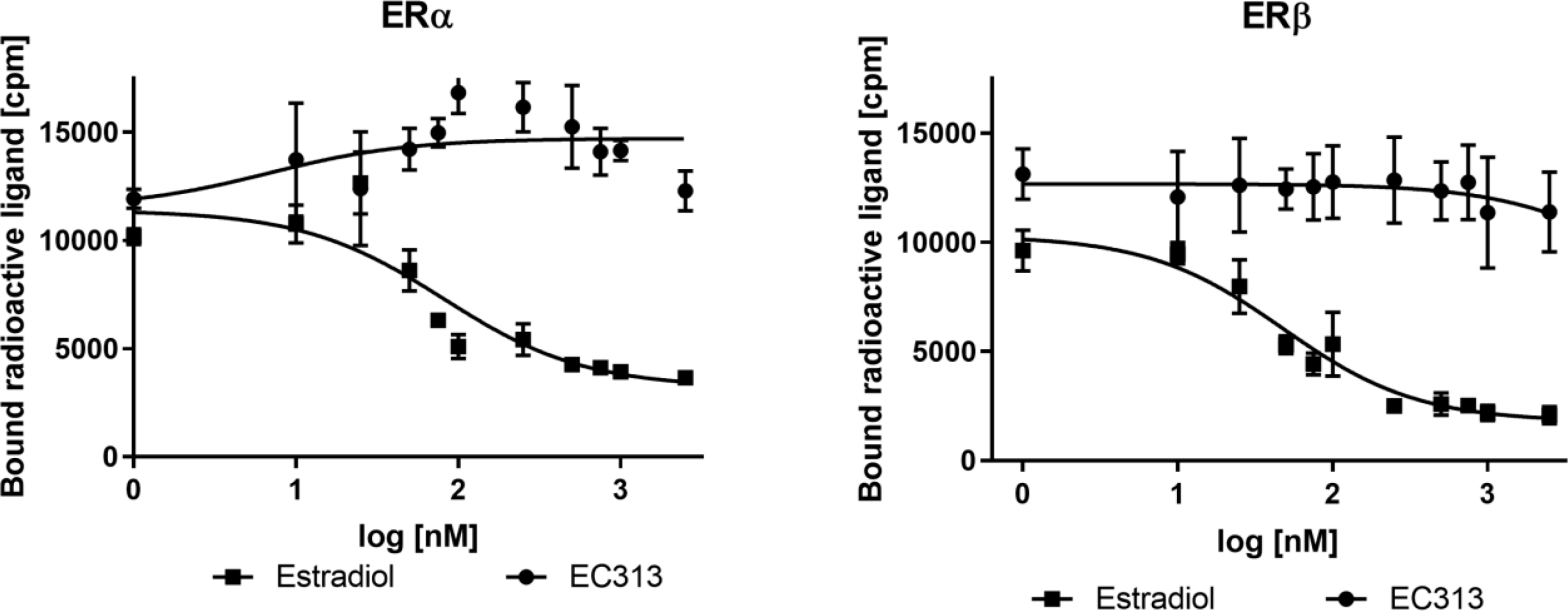
Competitive binding assay curve, binding of EC313 and Estradiol to ERα and ERβ.

There are two subtypes of estrogen receptors (ER), ERα and ERβ, coded in different genes. Plasmids pFastBac1-ERa and pFastBac1-ERb, coding for the full length human estrogen receptors α and β, respectively, were obtained from GenScript. Tritiated Estradiol served as the labeled reference. The competitive displacement was tested in three independent experiments for either receptor. EC313 did not cause any competitive displacement of [3H]-estradiol from either receptor class (**Figure 5**).

##### 4.1.1.5 Summary/conclusions of binding assays

The affinity of EC313 to the human progesterone receptor ligand binding domain is the same as progesterone. For the PR-A-isoform, the best agonistic activity can be obtained from stable transfection of hPR-A and a transient transfection for the MMTV-luciferase reporter gene. At the full-length PR- receptor isoform (PRA), EC313 is less potent with a competition coefficient of 3.4 compared to progesterone. A weak agonistic activity is identified with EC50 in the range of the maximum concentration tested (1 µM). As an antagonist, EC313 inhibits the Promegestone-induced reporter gene expression – the same as the reference antagonist Mifepristone. The potency of EC313 and Mifepristone are comparable (IC50 of 7.96 vs 6.19 nM, respectively). For the PR-B isoform, a sufficient transactivation signal was achieved using stable transfections for the human PR-B receptor and transient transfection for the MMTV-luciferase reporter gene. The results show that EC313 acts as an antagonist on the human PR-B receptor. In a set of 6 experiments, the average IC50 for EC313 was 2.5 ± 0.7 nM, and for Mifepristone 0.32 ± 0.06. The potency was, therefore, only approximately 13% of that of Mifepristone. EC313 also binds to the human glucocorticoid receptor though with lower affinity. Compared to Dexamethasone, the competition coefficient of EC313 was 2 at the ligand binding domain and 12 at the full-length receptor. At the human androgen receptor ligand binding domain, EC313 exhibited a competition coefficient of 17 compared to Metribolone (R1881). The attempts to express the full length receptor in insect cells did not yield cell lysates with a satisfactory specific binding. No competitive displacement of [3H]-estradiol was observed at both the human estrogen receptors α and β.

#### 4.1.2 Transactivation of human progesterone receptor

For PR-A, a weak agonistic activity was identified with EC50 in the range of the maximum concentration tested (1 µM).

**Figure 6:**
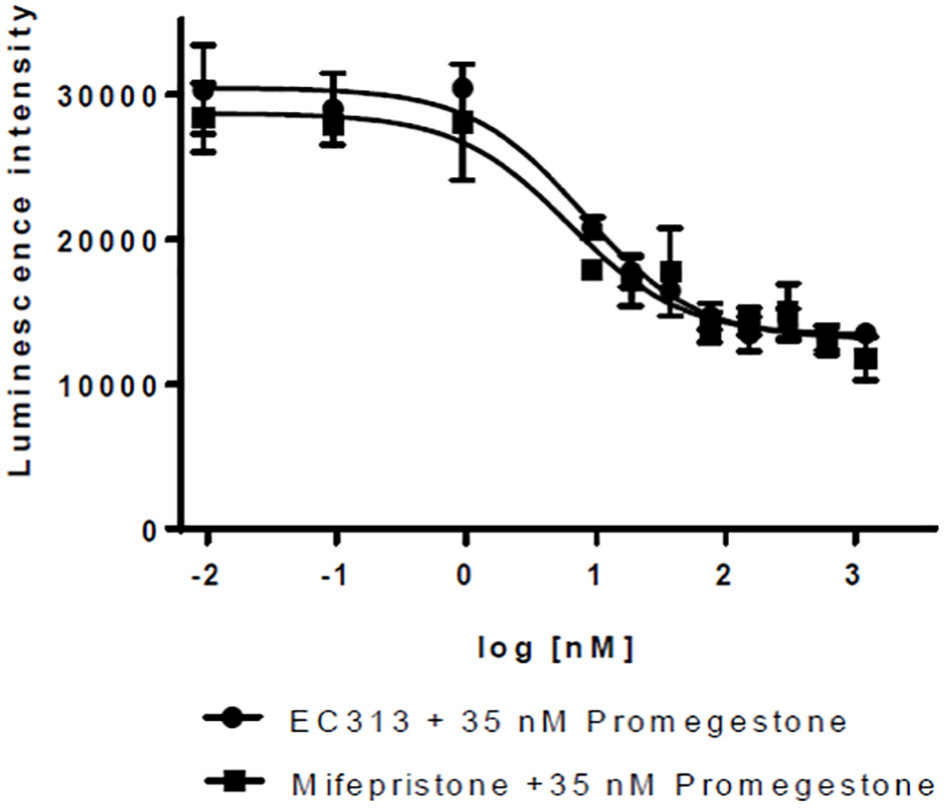
Transactivation dose response curves for EC313 on PR-a; EC313 shows full antagonistic activity.

As an antagonist, EC313 inhibited the Promegestone-induced reporter gene expression – the same as the reference antagonist Mifepristone. The potency of EC313 and Mifepristone were comparable (IC50 of 8.0 vs. 6.2 nM, respectively) (see **Figure 6**).

For PR-B, a sufficient transactivation signal was achieved using stable transfections for the human PR-B receptor and transient transfection for the MMTV-luciferase reporter gene.

**Figure 7:**
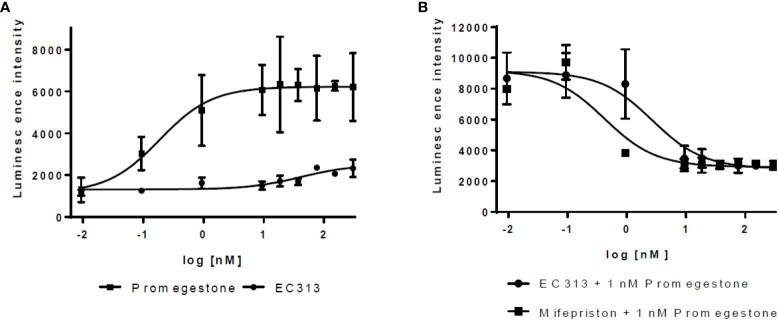
Transactivation dose response curves for EC313 on PR-B **(A)** agonistic activity, **(B)** antagonistic activity.

The results show that EC313 acts as an antagonist on the human PR-Breceptor. In a set of 6 experiments, the average IC50 for EC313 was 2.5 ± 0.7 nM, and for Mifepristone 0.32 ± 0.06. The potency was, therefore, approximately 13% of that of Mifepristone. Only marginal agonistic activity was recorded at high nM concentrations of EC313 (see **Figure 7**).

#### 4.1.3 Transactivation of human androgen receptor

**Figure 8:**
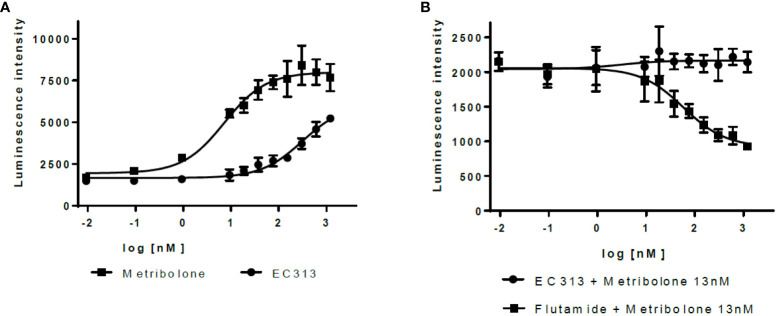
Transactivation dose response: AR **(A)** agonistic activity, **(B)** antagonistic activity.

EC313 demonstrates agonistic properties with low potency. Based on three experiments, EC313 had an EC50 of 280 nM compared to 7.3 nM for the reference Metribolone, i.e., the same androgenic effect requires a 38-fold higher concentration. No antagonistic activity was recorded (see **Figure 8**).

#### 4.1.4 Transactivation of human mineralocorticoid receptor

**Figure 9:**
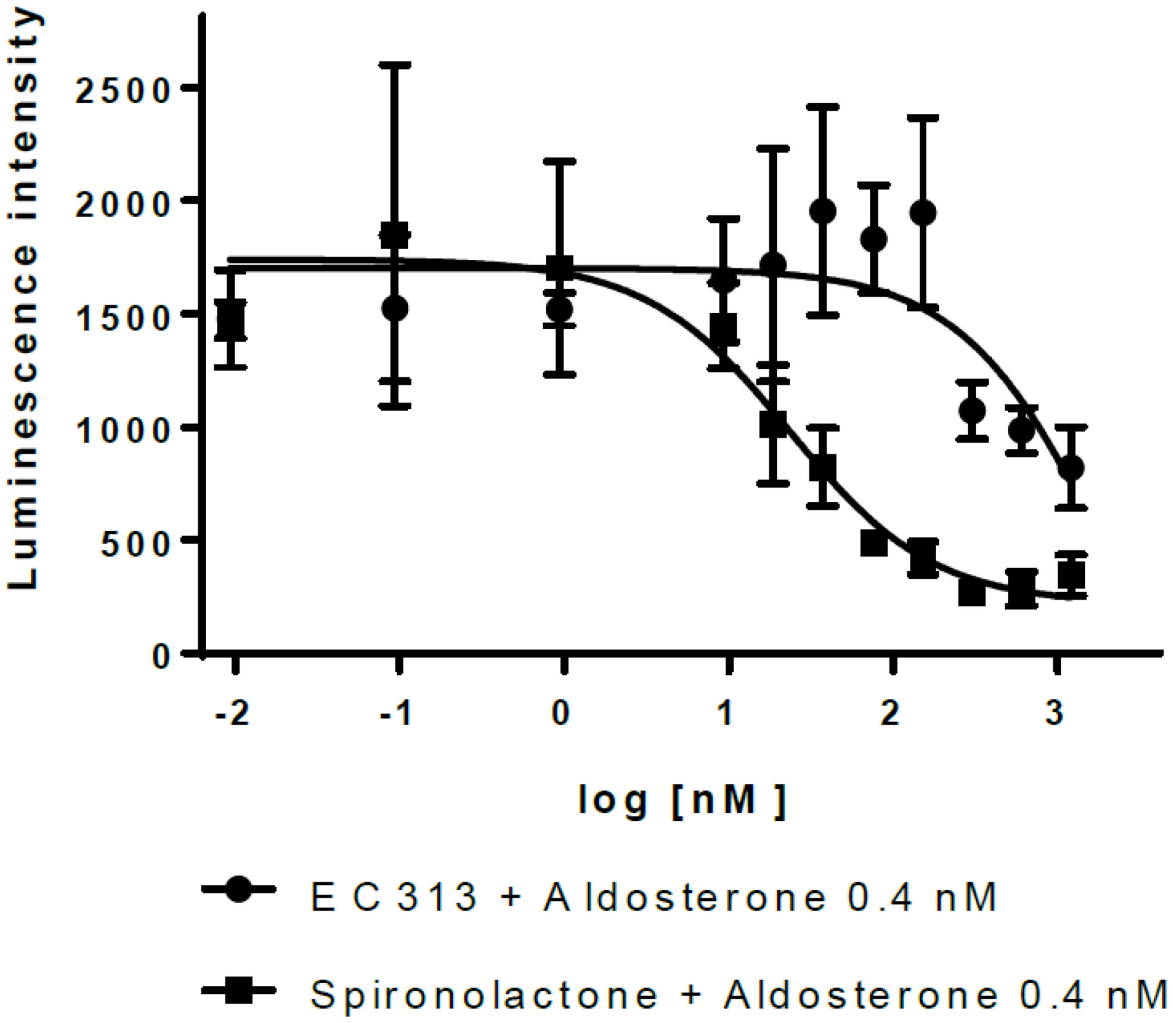
Transactivation dose response: MR antagonistic activity.

The transactivation data demonstrated that EC313 does not transactivate hMR. However, EC313 antagonizes transactivation induced by Aldosterone with low potency (IC50 2.0 µM), i.e., approximately 80-fold lower than Spironolactone (IC50 24 nM) (see **Figure 9**).

#### 4.1.5 Transactivation of human estrogen receptor

In the results for transactivation on human Erα, no agonistic or antagonistic activity was observed, which is in line with the observed lack of affinity to the human ER.

The ERβ transfected CHO cells—both after stable and transient transfection—behaved unreliable and gave unpredictable responses. In a control experiment, the estrogen-induced transactivation of the reporter gene was completely abolished by treatment with Mifepristone. This indicates that the control of the reporter gene expression is not ERß specific but influenced by PR or GR antagonists. Therefore, the results are not suitable for the characterization of potential ERß mediated effects of EC313, which is PR and GR antagonistic.

#### 4.1.6 Summary/conclusion of transactivation assays

The current report combines the experiments and results obtained in the program to analyze the transcriptional effects induced by EC313 on human steroid hormone receptors. While it was not possible to establish a specific assay for Erβ, this was considered of minor relevance in light of the lack of binding of EC313 to ER.

EC313 acts as an antagonist on both PRA and PRB. On the GR, EC313 acts as a weak inhibitor, whereas EC313 acts as a very weak agonist through the AR. In cells transfected with human Mineralocorticoid Receptor, an IC50 of 2 µM was estimated. The biological relevance of transactivation signals observed *in vitro* at concentrations that high may be rightfully questioned.

In conclusion, the transactivating properties of EC313 on human steroid hormone receptors reveal a profile in line with the properties of other SPRMs. The profile characterized for EC313 fits very well with that of reference SPRMs (**Figure 4**). In the transactivation assay employed, both androgenic and anti-glucocorticoid activity is quite common for that class of compounds.

The slight antagonistic activity of EC313-activated MR observed at the human MR with an IC50 of approximately 2000 nM was between 1% and 2.6% of Spironolactone. This effect was considered to be non-relevant for the *in vivo*-situation.

#### 4.1.7 Additional characterization of receptor activation

Progesterone receptors, when not activated, are located in the cytoplasm together with chaperone proteins. Upon binding to the ligand, they shed the chaperone proteins and are then capable of protein-protein interactions, nuclear translocation, binding to DNA-responsive elements, and interacting with and modulating gene transcription processes.

The following assays were performed to elucidate the signal pathways activated by the interaction of EC313 in comparison with other well-characterized progesterone receptor ligands.

##### 4.1.7.1 Induction of progesterone receptor nuclear translocation by EC313

**Figure 10:**
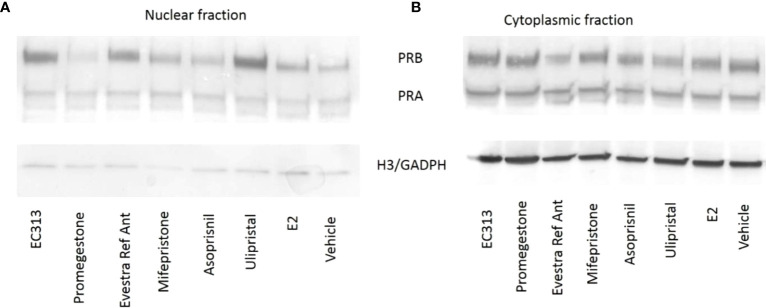
Nuclear translocation upon binding of PR-ligands to PRB and PRA. T47D cells were treated for 6h **(A)** and 24h **(B)** with 100 nM of PR-ligands in the presence of 100 pM E2. Loading controls: Histone H3 expression for nuclear extracts and GAPDH expression for cytosolic fractions.

To evaluate the ability of EC313 to translocate the intrinsic progesterone receptor to the cell nucleus, the human breast cancer cell line (T47D) was used. After the incubations, EC313, as well as all the other tested ligands, induced the presence of the progesterone receptor in the nuclear fraction. The extent of receptor degradation was ligand-dependent. At 24h, the staining was greatly reduced in the nuclear fraction without an increase in the cytoplasmatic fraction. This may indicate that the activated receptor undergoes enzymatic degradation. T47D cells exposed to EC313 or Ulipristal acetate retained more receptors in the nuclear fraction after 24 hours of stimulation when compared to Promegestone, Mifepristone, or Asoprisnil (**Figure 10**).

##### 4.1.7.2 Investigation of PR–B – NCOR interaction induced by EC313 using a two-hybrid system

**Figure 11:**
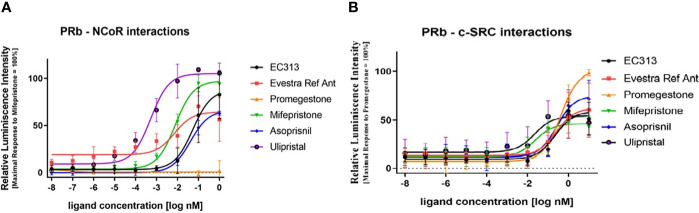
**(A)** PR-B interaction with NCoR using a two-hybrid assay. Dose response curve. The data presented are from five independent experiments and represented as mean relative luciferase units ± SD normalized to maximal mifepristone activity. **(B)** Liganded PR-Binteraction with c-SRC kinase. The data presented are from five independent experiments. Signals are represented as mean luminescence ± SD normalized to maximal promegestone activity equal to 100%.

The active recruitment of the nuclear corepressor NCoR results in transcriptional inactivity of the PR. So, the potency of PR ligands to induce the interaction of PR with NCoR provides information on the antagonistic potency of the ligand. Therefore, the capacity of EC313 to induce the interaction of PR-B with the corepressor NCoR in comparison with reference ligands was studied. All test compounds except the receptor agonist Promegestone induced PR to interact with NCoR. Ulipristal acetate was the most potent and efficacious inducer of the interaction with the nuclear co-repressor, followed by Mifepristone. The mesoprogestins Asoprisnil and EC313 demonstrated limited ability to induce progesterone receptor interaction with NCoR. Ranking the maximal efficacy of the test compounds to induce transcription of the reporter gene, which depends on the binding of PRB to NCoR, generates the sequence Ulipristal acetate > Mifepristone > EC313 > Asoprisnil, which is in good agreement with the pharmacology of the investigated progesterone receptor ligands (**Figure 11A**).

##### 4.1.7.3 Investigation of PR-B – c-SRC interaction induced by EC313 using a two-hybrid system

In this study, the ability of EC313 and the reference ligands (Promegestone, Mifepristone (RU486), Asoprisnil, and Ulipristal acetate) to enable the receptor–c-Src interaction was investigated. The tyrosine protein kinase c-SRC is involved in the regulation of cell proliferation, and activated nuclear steroid receptors are known to be able to interact with that protein kinase. All test compounds induced the interaction of PR with the c-SRC tyrosine kinase. SPRMs and mesoprogestins induced only 50% to 73% of the signal. The potency to induce the interaction was similar for Promegestone, the mesoprogestins, and EC317. Mifepristone and Ulipristal acetate were an order of magnitude more potent in eliciting the receptor–c-Src interaction.

Ranking the maximal efficacy of the test compounds to induce transcription of the reporter gene, which depends on the binding of PRB to NCoR, generates the sequence: Ulipristal acetate > Mifepristone > EC313 > Asoprisnil. In addition, Ulipristal acetate was the most potent of the test compounds. The potency and efficacy of the different ligands to induce the receptor interaction with NCoR are in good agreement with the pharmacology of the investigated progesterone receptor ligands:

- the agonist Promegestone does not induce NCoR interaction

- Ulipristal acetate is the most potent and efficacious inducer of the interaction with the nuclear co-repressor; Mifepristone follows in the ranking

- the mesoprogestins Asoprisnil and EC313 demonstrate a limited ability to induce progesterone receptor interaction with NCoR.

The assay confirms that all receptor ligands induce the c-Src interaction, though to varying extend and with different potencies, and consequently, have the potential to influence cell proliferation by this non-genomic signal cascade (**Figure 11B**).

The human nuclear receptor binding profile was in line with the expectations and confirmed good specificity for the human progesterone receptor. The binding of EC313 to the human glucocorticoid receptor might indicate some activity at that receptor. This would need to be confirmed *in vivo*. Binding to the human androgen receptor was weak but nevertheless deserves further *in vivo* studies. In contrast, EC313 is devoid of interactions with the estrogen receptor as no transcriptional activity was detected for EC313 on ER. The results are summarised in **Table 5**.

**Table 5:** Comparative transactivation profile.

SPRM	Potency [Nm]		
	PR Antagonism	GR Antagonism	AR Agonism
Ulipristal acetate	5, 3	95	n.a.
Vilaprisan	7, 0	629	96, 8
Asoprisnil	9, 0	1232	9, 0
EC313	2, 2	100	35, 4

The data convincingly demonstrate that EC313 binds to the human progesterone receptor, induces receptor translocation, and its binding to DNA-responsive elements. In addition, as the reference progesterone receptor ligands, EC313 induces the interaction of the human progesterone receptor with the tyrosine-kinase c-SRC. Moreover, EC313 induces the interaction of the receptor with NCoR (Nuclear Receptor Co-Repressor) and this suggests an antagonistic activity of EC313 at the receptor level. The interaction with NCoR points to its ability to inhibit receptor-induced transcription, though, with 200-fold lower potency and 83% of the efficacy of Ulipristal acetate. Thus, the potency of EC313 is similar to Asoprisnil. However, Asoprisnil represents only 56% of UPA efficacy. Remarkably, EC313 counteracts the estradiol-induced proliferation of T47D. This property confirms the nature of EC313 as a progesterone receptor modulator, not only with respect to the classical genomic signal cascade but also with respect to the non-genomic pathways (**Table 6**).

**Table 6:** Differential actions of EC313 and reference PR ligands on human PR.

Test Systems		Test Compounds					
Mechanism	Assay	R5020	Ulipristal Acetate	RU486	Asoprisnil	EC317	EC313
Classical	Nuclear Translocation	**+**	**+**	**+**	**+**	**+**	**+**
	DNA binding	**+**	**+**	**+**	**+**	**+**	**+**
	NCoR Interaction	**-**	**++**	**++**	**+**	**+**	**++**
Other Signal cascades	PR – cSRC	**++**	**+**	**+**	**+(+)**	**+**	**+**
	Activation of Cyclin D1 promotor	**+**	**+**	**+**	**+**	unclear*	**+**
Proliferation	Arrest in Go/G1**	**↓**	**↑**	**↑**	**↑**	**↑**	**↑**

* Results indicate a sticky behavior of EC317 in serial dilutions.

** Due to the variability of results in T47D cells, statistical significance was not always reached.

↓/↑ - decreased/increased action of tested compounds.

### 4.2 *In vivo* studies

#### 4.2.1 Guinea pig- studies

No fetal expulsions were observed with EC313 at 30 mg/animal/day. The tested dose of 30 mg/animal/day represents a 100-fold higher dose than the lowest anti-ovulatory effective dose of EC313 in cycling guinea pigs. UPA had no effect on pregnancy at the lowest tested dose (3.0 mg/animal/day s.c). But, higher doses (10.0 and 30.0 mg/animal/day s.c. respectively) induced expulsion of uterine contents in 1/5 and 3/5 animals, respectively.

Full inhibition of ovulation was observed at EC313 dosages of 1 and 10 mg/animal/day s.c, exceeding the effect of Ulipristal acetate (UA) or Mifepristone. In this context, regressing CLs can be explained by the PR-agonistic effects of EC313 at the uterine level. In this assay, EC313 showed effects in the vaginal epithelium (mucification) in non-pregnant guinea pigs at all tested dose levels, ranging from 0.1 to 10.0 mg/animal/day s.c. The absence of uterine growth, and in particular the morphologic transformation of the vaginal epithelium (mucification) may be attributed to a mesoprogestin. An increase in uterine mass was observed, which demonstrated that ‘pure’ PR-antagonists were not observed with the test compound EC 313. Estrogen receptor (ER) mediated adverse effects of this compound were not observed. The PR dominance prevents the unopposed estrogenic effect (see Elger et al. in press). Despite the highest s.c. dose (30 mg/animal/day), all the proprietary SPRMs used in the study (including EC313) showed no abortive potential in guinea pigs at the beginning of the last third of pregnancy (in contrast to Ulipristal acetate). The comparison of pure PR-antagonist (UPA) vs. mixed PR-agonistic/antagonistic mesoprogestins in guinea pig studies is seen as an option to reduce or abolish the labor-inducing properties of PR-modulating. At doses of 1.0 and 10.0 mg/animal/day s.c, the ovulation inhibition caused by EC313 was stronger than by mifepristone and UPA (**Table 7**).

**Table 7:** Comparison of the anti-ovulatory activities of reference SPRMs with that of EVESTRA SPRMs.

Dose (mg/animal/day s.c.)	Tested Compound						
	RU 486 Mifepristone	CDB 2914 UPA	CDB 4124 Telapristone acetate	BAY 1002670 Vilaprisan	EC312	EC313	EC317
0.1					1/5	1/5	2/3
0.3							3/3
1.0	4/5	3/3			0/5	0/5	0/3
3.0	2/5	5/6		0/5			0/6
10.0	0/3	2/6	1/3	0/4	0/8	0/8	0/6

Number of ovulations vs. number of inseminated females.

#### 4.2.2 The Influence of EC313 on estrogen-primed rabbit endometrium

**Figure 12:**
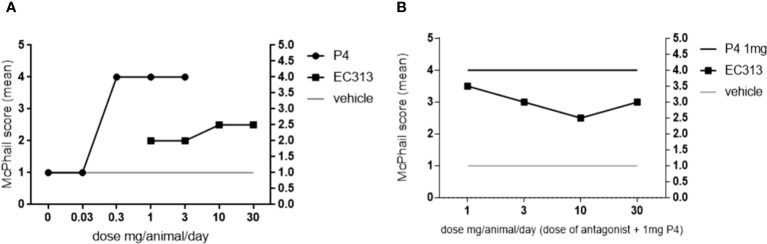
McPhail Test: Dose Response of Progesterone and EC313; agonistic **(A)** and antagonistic **(B)** progestogenic activity.

In the McPhail Assay, EC313 induced secretory endometrial transformation as reliable findings for a progestational action. However, the maximal effect did not exceed a McPhail score of 2.5 (average per treatment group). This indicates that the compound does not induce the full response of a progestin (McPhail score 4.0). The moderate “plateau-response” of agonistic action with failing of further dose-response relationship is typical for SPRMs (19). The co-administration of 1 mg/animal/day of progesterone and different doses of EC313 modified the effects of progesterone by gradually blunting the endometrial effects of progesterone and by inducing ‘atypical’ histology. At dosages of 10 and 30 mg/animal/day, the antagonistic action of EC313 was significant in comparison to other SPRMs (p< 0.05; Kuskal-Wallis- and Dunn-Test) (**Figure 12**).

**Table 8** below shows that in comparison with the first clinically investigated mesoprogestin, Asoprisnil, the compound EC313 possesses relatively more agonistic than antagonistic effects (approx. 7 times).

**Table 8:** Comparison of the McPhail results of EC313 with progesterone and other SPRMs.

Compound	Score 2 for PR-antagonistic activity (mg/animal/day s.c.).	Score 2 for PR-agonistic activity (mg/animal/day s.c.)	Quotient between antagonistic and agonistic action
Progesterone	n.a. “pure” agonist	~ 0.1	n.a.
Vilaprisan	< 0.3	n.a.;”pure” antagonist	n.a.
Asoprisnil	~ 0.3	~ 0.2	~ 1.5
EC313	~ 10.0	~ 1.0	~ 10.0*

*the relative share of EC313 PR activity.

The rating scale ranges from 1 (no or very low secretory, transformation, or agonistic activity) to 4 (highest secretory, transformation, or agonistic activity). The McPhail score of 2 (median) was selected for the comparison. The antagonistic activity was measured by the concomitant administration of progesterone (1 g/animal/day s.c.).

#### 4.2.3 Glucocorticoid/antiglucocorticoid activities

**Figure 13:**
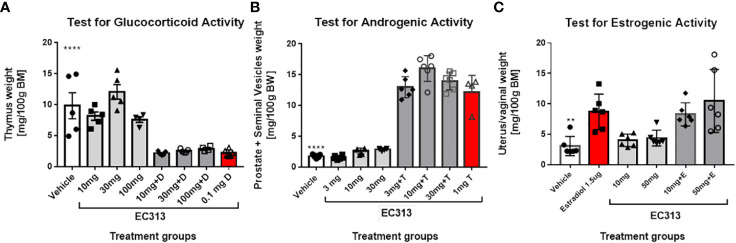
**(A)** Thymus involution test in adrenalectomized rats – thymus weight in control and treatment groups of animals. Data are presented as mean ± SD. Statistically significant ****P<0.0001, the comparison was done vs. vehicle (negative control) and Dexamethasone (D). **(B)** Results of the Hershberger Test for androgenic and anti-androgenic effects in rats. Animals were treated daily with the indicated doses of EC313 or EC313 + Testosterone propionate (T). The testosterone propionate dose was 1 mg/ animal /day. Negative control rats received vehicle only. Results for vehicle-treated animals differ significantly from all groups receiving T (****), p<0.0001. **(C)** Results of the Uterus-Growth-Assay in Immature Female Rats (Estrogenicity/Anti-Estrogenicity).

In the Standard Thymus-Involution-Test in rats, a reduced weight of the thymus indicates glucocorticoid activity. The abolishing of the Dexamethasone-induced thymus reduction by the test compound indicates anti-glucocorticoid activity. EC313 has neither glucocorticoid nor anti-glucocorticoid activities at doses up to 100 mg/animal/day s.c. (**Figure 13A**).

Statistical evaluation was performed using the Mann-Whitney test. In contrast to the *in vitro*-findings recording the transactivation activity of EC313 on GR, *in vivo*, no glucocorticoid or anti-glucocorticoid action was seen.

#### 4.2.4 Androgenic or anti-androgenic properties

The Hershberger Assay was used for the determination of androgenic as well as anti-androgenic properties. Statistical evaluation was performed using the Mann-Whitney test. Despite an increase in the weights of the prostates plus seminal vesicles under treatment with 10 mg EC313/animal/day, neither significant androgenic nor anti-androgenic effects were observed (**Figure 13B**).

#### 4.2.5 Estrogenic and anti-estrogenic activities of EC313 in the uterus growth test in rats

EC313 did not induce a significant increase in the weight of uterine horns and vaginal weights vs. vehicle-treated rats. Similarly, co-administration of EC313 to E2 did not significantly reduce the weight of uterine horns and vagina when compared to those treated with E2 alone. In conclusion, the results indicate neither estrogenic nor anti-estrogenic activity of EC313 at daily extremally high s.c. doses of up to 5mg/animal corresponding to 172 mg/kg body weight in rats (**Figure 13C**).

#### 4.2.6 Human endometriotic tissue transplantation into immunodeficient mice

Compared with other preclinical models, the chosen method with human transplants remains one of the most suitable models.

**Figure 14:**
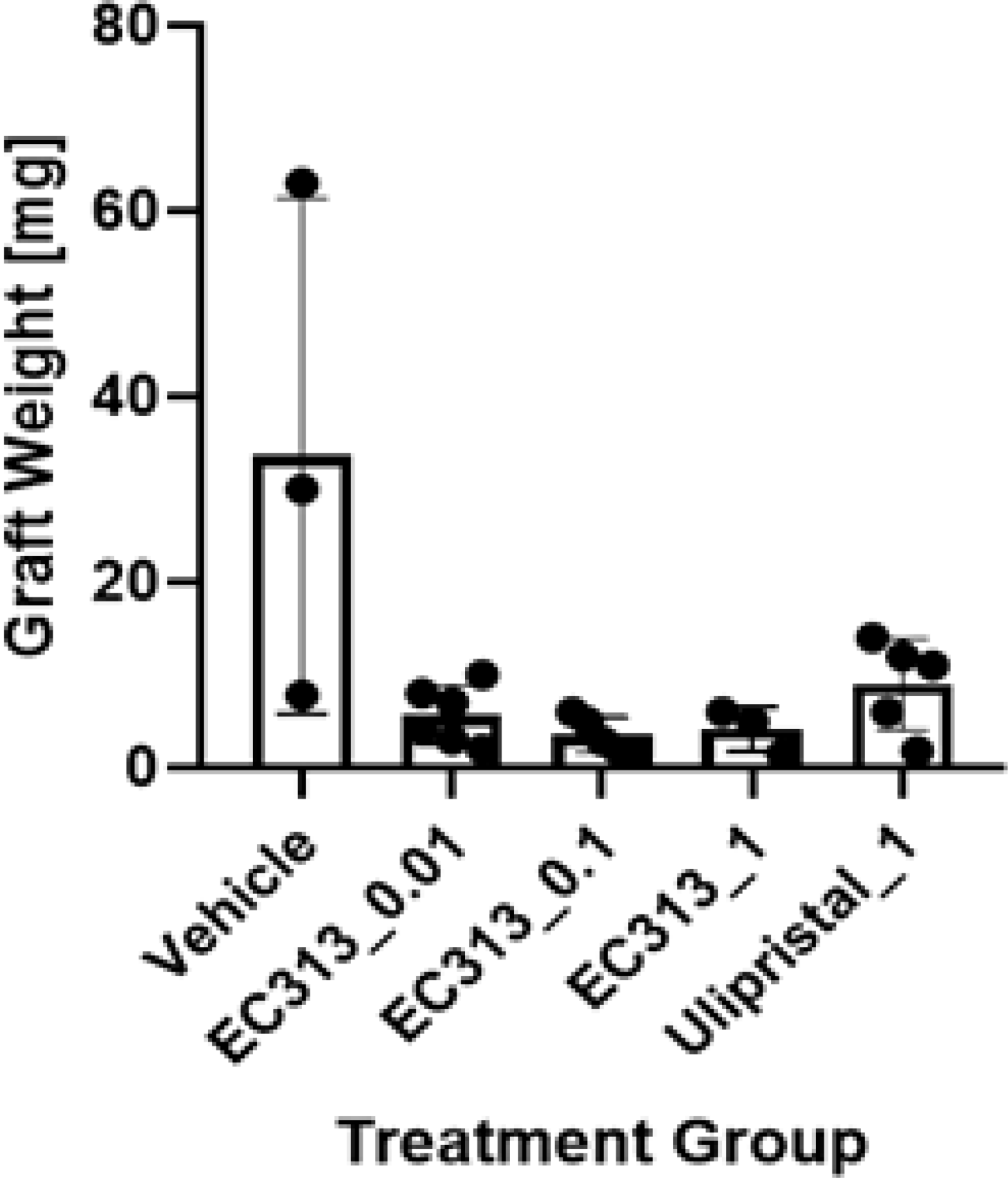
The effect of EC313 on human endometriotic tissue in subcutaneous pouches of immunodeficient mice. Weights of human endometriotic tissue grafts at the end of the experiment (mean and individual values). Treatment group labels indicate the test compound and daily dose [mg/animal/day]. Grafts were detected in between 3 and 6 mice per treatment group.

EC313, and to a lower extent the Ulipristal treatments, resulted in significantly lower weights of the grafts compared to vehicle control, indicating an anti-endometriotic effect. Therefore, EC313 showed better anti-endometriotic activities than UPA (**Figure 14**).

#### 4.2.7 Human uterine fibroid tissue transplantation into immunodeficient mice

**Figure 15:**
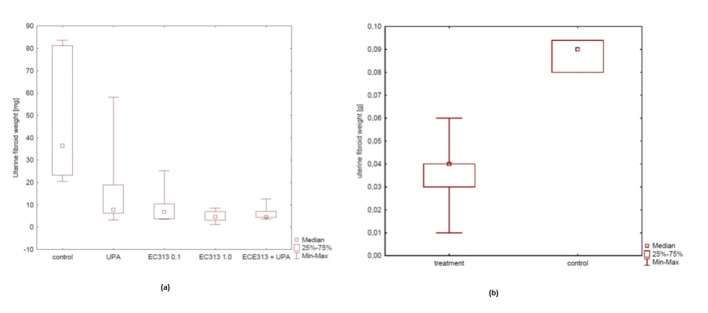
**(A)** Influence of EC313 at different dosages (0.1 and 1.0 mg/kg/day), UPA, and UPA plus EC313 (UPA (5.0 mg/kg/day) on the weight of human fibroid xenografts in immunodeficient mice. Duration of s.c. treatment: 60 days; treatment for 5 days per week (Part A). **(B)** Comparison of average fibroid weight between the control group and the very low dose treated EC313 group (0.01 mg/kg/day) (Part B).

Fibroids/Leiomyoma are one of the expected clinical target indications for the development of EC313[2]. But, there are no very reliable pre-clinical models at present. From all experimental approaches, the s.c. transplantation of human fibroid tissue into immunodeficient mice resembles the clinical situation most closely. The anti-fibroid activity of EC313 at low dosages was better than that of UPA in this model and was confirmed in two independent studies (**Figure 15**). C313 treatment reduced the fibroid xenograft weight dose-dependently (p<0.01). When compared to the control animals, E2-induced proliferation was blocked significantly in the EC313-treated xenograft fibroids (p< 0.0001). The anti-fibroid activity of EC313 was significantly higher than that of UPA. When given simultaneously with UPA, EC313 further reduced the xenograft weight. The uterine weight was reduced when compared to UPA, indicating lower estrogenic effects. Concerning the histological findings, human fibroids preserved the fibroid histology of collected samples. After EC313-treatment, the levels of ER and PR were reduced, as shown by immunohistochemistry vs. control (p< 0.001) and with UPA (p< 0.01). The uterine fibroid markers such as desmin and collagen were markedly reduced upon treatment with 0.1 and 1.0 mg EC313/kg/day (Part A). The anti-fibroid activity of EC313 was also demonstrated with the 10 times lower dosage of 0.01 mg/kg/day (Part B).

## 5 Discussion

The affinity of EC313 to the human progesterone receptor ligand binding domain is the same as progesterone. At the full length PR-receptor isoform (PRA), EC313 was less potent with a competition coefficient of 3.4 compared to progesterone. A weak agonistic activity was identified with EC50 in the range of the maximum concentration tested (1 µM). As an antagonist, EC313 inhibits Promegestone (strong progestin) induced reporter gene expression completely – similar to the reference antagonist Mifepristone.

On the other hand, EC313 acts as an antagonist on the human PR-B receptor. But, the potency was approximately only 13% of that of Mifepristone. The clinical consequences of this shift in the PRA/PRB- quotient including progesterone resistance in endometriosis still need to be clarified (7, 8, 20–23).

As shown in several experiments on further molecular mechanisms, EC313 interacted on the human PR in the frame of the classical signal cascades (**Table 5**). *In vitro*, EC313 also bound to the human glucocorticoid receptor (GR) with lower affinity. At the human androgen receptor ligand binding domain, EC313 exhibited a competition coefficient of only 17 compared to the reference androgen Metribolone (R1881).

No competitive displacement of [3H]-estradiol was observed at both, the human estrogen receptor α and β. However, several *in vivo* tests in rats did not show estrogenic/anti-estrogenic, androgenic/anti-androgenic, and glucocorticoid/anti-glucocorticoid activities.

Regarding the *in vivo* studies, which are important for the pharmacodynamic or the expected therapeutic profile of EC313, the guinea pig model showed the same features for termination of pregnancy by classical and “pure” antiprogestins such as Mifepristone as also demonstrated in humans. It is possible to distinguish between progestational and anti-progestational activities in the same experiment. In guinea pigs, EC313 showed mixed agonistic as well as antagonistic activities at the progesterone receptor (PR). This profile fulfills the criteria of mesoprogestin. The EC313 treatment revealed PR-dominance at the genital tract and inhibited unopposed estrogenic effects. In the very high dosage of 30 mg/animal/day s.c. (approx. equivalent to 60 mg/kg/b.w.) given twice on pregnancy days 43 and 44, no premature labor was induced (in contrast to UPA which was dosed at 10 and 30 mg/animal/day s.c.). It is important to note that the anti-ovulatory activity of EC313 in guinea pigs (0.1-1.0mg/animal/day s.c.) exceeded that of Ulipristal or Mifepristone (RU 486).

The endometrium represents the most sensitive target tissue for SPRMs (24). Therefore, the McPhail assay for the determination of PR-agonistic and/or PR-antagonistic activities on the estrogen-primed rabbit endometrium is a key method for the pharmacological/endocrinological characterization of a given SPRM. The results of the McPhail assay with progestins correlate with the findings in humans (25). In this test regime, EC313 showed moderate PR-agonistic and low but significant PR-antagonistic activities. The ratio between agonistic and antagonistic properties was much higher in comparison to the other clinically investigated mesoprogestin Asoprisnil (see **Table 8**).

The preclinical assays for the intended clinical indications are the two tests with humanized uterine fibroid and endometriotic xenografts in mice. To our knowledge, no better reliable preclinical models for the reflection exist for the two human disorders uterine fibroids (leiomyomata uteri) and endometriosis. In these studies, EC313 in the low dose range acts stronger than the reference SPRM UA, which is approved for the treatment of heavy menstrual bleeding caused by uterine fibroids. In addition, several clinical studies demonstrated the shrinkage of uterine fibroids and endometriotic lesions by Ulipristal acetate (12). It remains noticeable that the dosages for obtaining pharmacological effects in guinea pigs (vulva, endometrium, ovulation) and rabbits (endometrium) starting with 3.0 mg/kg or 1 – 10 mg/kg s.c.) are clearly higher than that for the treatment of fibroid- and endometriotic grafts in mice, starting with 0.01 mg/kg s.c. These differences can be based on species differences or the tissue-specificity of a bifunctional principle. Another explanation could be the possibility that the mixed agonistic/antagonistic feature of EC313 is target-specific in terms of a superadditive. It is well documented that progestins themselves show therapeutic efficiency against endometriosis as well as fibroids (26, 27). Therefore, it can be assumed, that in the case of EC313, the combination of clear but dose-dependent limited PR-agonistic and moderate but significant PR-antagonistic activities can explain the superiority of this compound in the clinically relevant models for endometriosis and fibroids using humane xenografts. In this regard, there is a significant difference in the mode of action between EC313 and other SPRMs, including Asoprisnil.

In conclusion, the new mesoprogestin EC313 is a promising candidate for original approaches in the treatment of fibroids, endometriosis including adenomyosis, heavy uterine bleeding, and other disorders in the field of women’s health. EC313 shows a unique ratio between PR-agonistic and PR-antagonistic activities. It is highly effective in humanized *in vivo* models for endometriosis and fibroids.

## Data availability statement

The original contributions presented in the study are included in the article. Further inquiries can be directed to the corresponding author.

## Ethics statement

The animal studies were reviewed and approved by Local Ethics Committees.

## Author contributions

KB-S: conceptualization of the studies, concept implementation and *in vitro* and *in vivo* studies conduction, data analysis, and manuscript supervision. AK and EM: studied conduction and development. WE: development of mesoprogestins concept, conceptualization and its *in vivo* implementation. PS: conducted the surgeries in order to obtain endometriosis samples. MO: supervised the studies and wrote the manuscript. NH conducted uterine fibroids studies. MW: is the project leader and controller for the EU Project.
